# Evidence of Eelgrass (*Zostera marina*) Seed Dispersal by Northern Diamondback Terrapin (*Malaclemys terrapin terrapin*) in Lower Chesapeake Bay

**DOI:** 10.1371/journal.pone.0103346

**Published:** 2014-07-29

**Authors:** Diane C. Tulipani, Romuald N. Lipcius

**Affiliations:** Fisheries Science, Virginia Institute of Marine Science College of William & Mary, Gloucester Point, Virginia, United States of America; Università della Calabria, Italy

## Abstract

The initial discovery in May 2009 of eelgrass (*Zostera marina*) seeds in fecal samples of wild-caught northern diamondback terrapins (*Malaclemys terrapin terrapin*) was the first field evidence of eelgrass seed ingestion in this species. This finding suggested the potential of terrapins as seed dispersers in eelgrass beds, which we sampled for two additional years (2010 and 2011). Seeds were only found in feces of terrapins captured prior to June 8 in all three years, coinciding with eelgrass seed maturation and release. Numbers of seeds in terrapin feces varied annually and decreased greatly in 2011 after an eelgrass die off in late 2010. The condition of seeds in terrapin feces was viable-mature, germinated, damaged, or immature. Of terrapins captured during time of seed release, 97% were males and juvenile females, both of which had head widths <30 mm. The fraction of individuals with ingested seeds was 33% for males, 35% for small females, and only 6% for large (mature) females. Probability of seed ingestion decreased exponentially with increasing terrapin head width; only males and small females (head width <30 mm) were likely to be vectors of seed dispersal. The characteristic that diamondback terrapins have well-defined home ranges allowed us to estimate the number of terrapins potentially dispersing eelgrass seeds annually. In seagrass beds of the Goodwin Islands region (lower York River, Virginia), there were 559 to 799 terrapins, which could disperse between 1,341 and 1,677 eelgrass seeds annually. These would represent a small proportion of total seed production within a single seagrass bed. However, based on probable home range distances, terrapins can easily traverse eelgrass meadow boundaries, thereby dispersing seeds beyond the bed of origin. Given the relatively short dispersion distance of eelgrass seeds, the diamondback terrapin may be a major source of inter-bed seed dispersal and genetic diversity.

## Introduction

Plants rely on abiotic and biotic processes by which to transport their seeds to suitable habitat [1]. Some plants have adaptations for abiotic dispersal that slow the rate of descent, yet maximize horizontal distance [1]. Biotic dispersal relies on other organisms to move seeds to new locations, often further than abiotic processes can achieve [1]. Seed acquisition and transport can be realized through active or passive involvement of the organism [1]. Seed ingestion is a passive mode of dispersal and can result from mutualism between plants and animals [1]. Dispersal by animals has been well studied, yet categorizing whether or not a species is an effective disperser can be challenging. Effective biotic dispersal can be critical to a plant's reproductive success [2] and depends on the number of seeds consumed and egested, as well as the probability that a dispersed seed will germinate in the new habitat [2].

Saurochory is the dispersal of plants by reptiles [3] and is defined specifically for turtles as chelonochory [4]. Many chelonian species of varying foraging strategies ingest seeds, though most are herbivorous [5], [6]. For instance, terrestrial species that ingest terrestrial seeds include the Galápagos tortoise *Chelonoidis nigra* [7], Florida box turtle *Terrapene carolina bauri* [8], and the Amazonian tortoise *Geochelone denticulata* [9]), aquatic species ingesting aquatic and terrestrial seeds include the black river turtle *Rhinoclemmys funerea* [3], and aquatic species ingesting terrestrial seeds include the red-eared slider *Trachemys scripta elegans* and the common snapping turtle *Chelydra serpentina* [10].

Most aquatic chelonid dispersers occur in freshwater. Few reptiles are adapted to living in salt water and even fewer are turtles [11]. Of the seven marine turtle species, green sea turtles *Chelonia mydas* are well-known consumers of turtle grass *Thalassia testudinum*, yet their potential as seed dispersers is unknown [12]. In North America, the diamondback terrapin *Malaclemys terrapin* is the only aquatic and fully estuarine species of turtle [6]. *Malaclemys* inhabits salt marshes from Massachusetts to Texas, and forages in seagrass beds in lower Chesapeake Bay [13]. The northern portion of the terrapin's range between the Outer Banks of North Carolina to Cape Cod, Massachusetts overlaps with the distribution of a primary temperate species of seagrass, eelgrass *Zostera marina* [6], [14], which is the dominant seagrass species in Chesapeake Bay [15]. Small terrapins prefer shallow, near-shore brackish water regions of estuaries and coastal bays [13], [16], where eelgrass meadows commonly occur in lower Chesapeake Bay and the coastal bays of Virginia's Eastern Shore peninsula [14].

In May 2009, eelgrass seeds were found among pieces of eelgrass leaves and remains of eelgrass epifauna and benthic fauna in fecal samples from diamondback terrapins captured in the lower York River, Virginia [13]. At the time, it was unknown whether or not the seeds were incidentally ingested [13]. Prior to this finding, only waterfowl and several fish species had been considered as biotic dispersal agents for eelgrass in temperate habitats [17]–[19] though dispersal distances for fishes were small and timing of seasonal foraging by waterfowl on seagrasses was incongruous for seed dispersal [17], [20]. Because of physical characteristics of eelgrass seeds, nearly all seeds remain in the bed of origin despite abiotic processes that could transport the seeds greater distances [21]. Long-distance abiotic dispersal of floating eelgrass seed pods (spathes) can result in colonization of new seagrass beds [22]. Finding that diamondback terrapins ingested eelgrass seeds raised the question of whether terrapins could be a vector for seed dispersal both within and between seagrass beds.

The aim of this study was to evaluate the role of diamondback terrapins in eelgrass seed dispersal in lower Chesapeake Bay. Specifically, we aimed to answer the following questions:

1. What is the frequency of occurrence of ingested *Zostera marina* seeds in fecal material of the northern diamondback terrapin *Malaclemys terrapin terrapin*?
2. Is seed ingestion based on diamondback terrapin size and habitat use?
3. Are egested seeds viable?
4. Are field-collected seeds viable and capable of germination?
5. What is the potential for seed dispersal by diamondback terrapins?

## Materials and Methods

**Figure 1:**
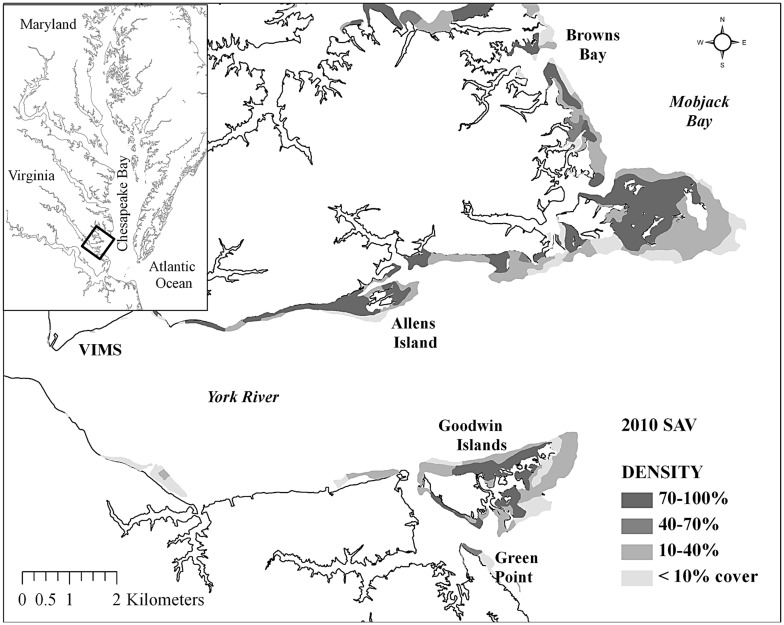
Diamondback terrapin collection locations from southwestern Chesapeake Bay SAV beds. Collection locations for diamondback terrapins from SAV beds of (A) Allens Island, (B) Goodwin Islands, and (C) Green Point along the lower York River subestuary and from (D) Browns Bay in southeastern Mobjack Bay, southwestern Chesapeake Bay (rectangle on inset). Modified from Orth et al. 2011.

Diamondback terrapins were collected from submerged aquatic vegetation (SAV) beds adjacent to Goodwin Islands, Green Point, and Allens Island along the York River subestuary, and in Browns Bay from May to early June in 2009, 2010 and 2011 (Figure 1), as part of a diet study [13]. Most terrapins were captured using a 4.9 m trawl, though some were captured by hand, bottom scrape, or commercial crab pots modified to prevent drowning. Captured terrapins were marked with a unique turtle identification number (TID) etched into marginal scutes along the right side of the carapace [23] plus one additional notch made in the second to the left, rear marginal scute to distinguish these captures from previous collections. Gender and standard morphological measurements for turtles were recorded, including head width (HW), straight carapace length (CL), plastron length (PL), and mass. Gender was determined by external characteristics of tail length and cloacal vent position with respect to the posterior edge of the carapace. Males have longer, thicker tails with the cloacal vent beyond the edge of the carapace [6]. Terrapins were grouped into two size classes based on head width, as small (HW<30 mm) and large (HW≥30 mm). All were released at the original collection location.

### Ethics Statement

Diamondback terrapin collection was authorized under Virginia Department of Game and Inland Fisheries scientific collection permits 034390 in 2009 and 038407 in 2010 and 2011, as well as Virginia Marine Resources Commission permits 09–012, 10–024, and 11–050 for 2009, 2010, and 2011, respectively. The Chesapeake Bay National Estuarine Research Reserve in Virginia granted a scientific research permit from 2009 through 2011 to sample at the Goodwin Islands. This study was carried out in strict accordance with the recommendations in the Guide for the Care and Use of Laboratory Animals of the National Institutes of Health. Two three-year protocols, IACUC-2008-07-17-5364-rnlipc and IACUC-2011-08-05-7415-rnlipc, were approved by, and renewed annually with, the College of William & Mary's Institutional Animal Care and Use Committee.

### Collection and viability of eelgrass seeds ingested by terrapins

In 2009, each captured terrapin was placed in a separate bucket with freshwater and returned to a laboratory at the Virginia Institute of Marine Science (VIMS). Fecal material egested by each terrapin during transit to the lab was collected upon return. Terrapins were housed individually in aquaria for 3–5 d and were not fed during this period [13]. To stimulate defecation, terrapins were kept in fresh water and items were collected from fecal material [24]. Fecal samples were rinsed with freshwater through a 1-mm standard test sieve, condensed into small pre-weighed drying trays, and air-dried prior to sorting.

Discovery of dry eelgrass seeds in dried samples in June 2009 prompted the change to brackish water in the fecal material collection protocol for 2010 and 2011. From capture through fecal material collection, terrapins were kept in brackish water from the York River to maintain potentially viable egested eelgrass seeds. These samples were rinsed with brackish river water through the sieve, collected into drying trays, and then checked for presence of eelgrass seeds. For each sample in which seeds were found, the seeds were removed and stored in brackish water in individual glass vials.

Analysis of variance, ANOVA (α = 0.05), was used to test for statistical significance of number of eelgrass seeds ingested by terrapins by gender or year collected. Presence of ingested seeds was analyzed using logistic regression to determine which factor (i.e., terrapin gender, head width, or year, plus interaction of gender and head width) best predicted ingestion of eelgrass seeds. Using a generalized linear model (GLM) with binomial distribution, nine candidate models were compared using Akaike Information Criterion with small sample correction (AIC_c_) to select the most parsimonious model [25].

In 2010 and 2011, 28 and 19 ingested seeds, respectively, were checked for viability. A seed was deemed viable if it was firm when gently squeezed with a pair of forceps. This criterion was tested for germination rate with the field-collected seeds, as described below.

### Field abundance of eelgrass seeds

**Figure 2:**
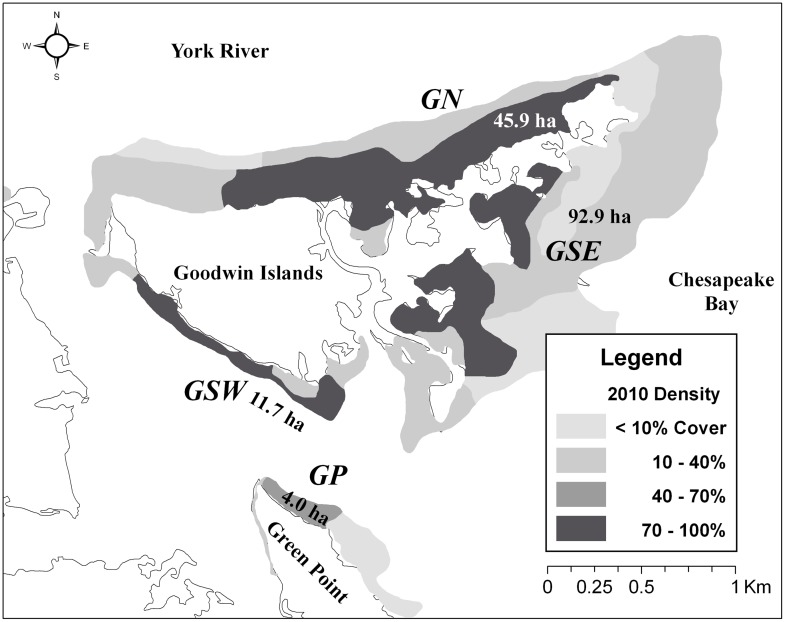
Regions in Chesapeake Bay where eelgrass samples were collected in May 2010. Regions with area (ha) where eelgrass samples were collected in May 2010 from SAV beds adjacent to Goodwin Islands (GN, GSW, GSE) and Green Point (GP) with percent coverage ≥40% (modified from Orth et al. 2010). GSE area included two coves with >70% cover.

To estimate eelgrass reproductive shoot and seed abundances in local SAV meadows, 72 random samples were collected during peak reproductive shoot biomass and seed development in May 2010 from SAV beds with at least 40% SAV cover in three regions of the Goodwin Islands; i.e., north (GN), southeast (GSE), and southwest (GSW), and from Green Point (GP) (Figure 2) [15]. Samples were collected in 1-mm mesh bags and stored by region in separate outdoor holding tanks with flow-through brackish river water until processed for reproductive shoot removal and seed counts.

Reproductive shoots from each replicate were bagged separately and frozen to estimate number of spathes per shoot and number of seeds per spathe. Mean abundance per m^2^ of reproductive shoots, spathes, and seeds were estimated for each region and week sampled and compared using ANOVA (α = 0.05). Linear regression was used to predict eelgrass reproductive shoot abundance as a function of region and week sampled. Four candidate models were compared using AIC_c_ to select the most parsimonious model [25].

### Viability of field-collected eelgrass seeds

By mid-June 2010, seeds were retrieved from each holding tank and stored in jars of York River water. A minimum of 10% of seeds collected from each holding tank was tested for viability, which was determined using the tetrazolium chloride staining method [26]. A seed was deemed viable if the embryo was stained pink after 24 h. Percent viable was calculated as the number of pink-stained embryos divided by the total number of seeds.

### Germination of field-collected seeds

In 2010 and 2011, potentially viable field-collected seeds were stored for approximately 6 mos until ambient water temperature was less than 15°C. [Eelgrass seeds germinate *in situ* in anoxic sediment when water temperature is near 0–10°C] [27]. Following experimental design described in Sumoski and Orth [20], 20 non-ingested, viable eelgrass seeds (supplied by the VIMS Seagrass Ecology Lab) were planted in individual containers at approximately 0.5 cm depth in sieved sediment from the York River. As before, a seed was deemed viable if it was firm when gently squeezed with a pair of forceps. All containers were placed in an outdoor tank with flow-through brackish river water. Containers were checked daily for visible leaf parts above the sediment, which indicated germination and plant growth. Seeds collected in 2010 were left in the flow-through tank until April 2011, at which time all cups were removed and checked for seed germination. The planted seeds collected in 2011 were removed in April 2012. Germination rate was calculated as number of seeds germinated divided by total number of seeds planted. Fisher exact test (two-sided) was used to test whether or not the ratio of germinated:planted seeds was different from 1∶1.

## Results

### Frequency and viability of eelgrass seeds ingested by terrapins

Over all three years, 118 terrapins were captured from early May through early June and examined for seeds; no seeds were found in fecal material from terrapins captured after June 8 each year. Of the 118 terrapins, 92% had ingested pieces of eelgrass leaves, which indicated foraging within the SAV beds [13]. The highest occurrence of ingested eelgrass seeds was in small terrapins (HW<30 mm) of both sexes: 33% of small males and 35% of small females with little interannual variation (Table 1). In contrast, only 6% of large females had ingested eelgrass seeds (Table 1).

**Table 1:** Number of diamondback terrapins captured May-early June 2009, 2010, and 2011 by gender and size class, and number that egested eelgrass (*Zostera marina*) seeds from SAV beds from regions Allens Island (1), Browns Bay (2), Green Point (3), Goodwin Islands (4), and Perrin Cove (5).

Year Gender (Size Class)	Regions	Captured	With Seeds	Percent with Seeds	Live Seeds	Dried Seeds	Total Seeds
2009							
males (s)	1, 3, 4	17	7	41%	0	34	34
females (s)	1, 3	3	1	33%	0	1	1
females (L)	4	1	0	0%	0	0	0
Total		21	8	38%	0	35	35
2010							
males (s)	1, 3, 4	26	11	42%	12	3	15
females (s)	1, 3	11	4	36%	8	5	13
females (L)	3, 5	2	0	0%	0	0	0
Total		39	15	38%	20	8	28
2011							
males (s)	All	40	9	23%	11	3	14
females (s)	2, 3	3	1	33%	2	0	2
females (L)	1, 2, 3, 4	15	1	7%	3	0	3
Total		58	11	19%	16	3	19
2009–2011							
males (s)	All	83	27	33%	23	40	63
females (s)	1, 2, 3	17	6	35%	10	6	16
females (L)	All	18	1	6%	3	0	3
Total		118	34	29%	36	46	82

Mean (±SE) of CL  =  straight carapace length; HW  =  head width; Mass; s: HW <30 mm; L: HW ≥30 mm.

By early June 2009, 35 seeds had been found in dried fecal samples. An additional 28 and 19 seeds were found in 2010 and 2011, respectively (Table 2). The number of seeds ingested per terrapin averaged 2.4 (SE  = 0.1) with a maximum of 13 by one male. Of 18 large females (HW ≥30 mm), only one had ingested eelgrass seeds (Table 1), though pieces of eelgrass were in fecal material from all but four [13]. The most “*in situ*-ingested” seeds were collected from terrapins in the Green Point region (Figure 2).

**Table 2:** Condition of eelgrass (*Zostera marina*) seeds in fecal samples from terrapins captured in SAV beds from May-early June 2010 and 2011.

Year*	Collected Seeds	Potentially Viable Seeds	Immature Seeds	Damaged Seeds	Germinated Seeds	Dead Seeds
2010	28	11	7	1	1	8
2011	19	5	7	1	1	5

*Thirty-five seeds found in 2009 were dried before discovered and could not be tested.

**Figure 3:**
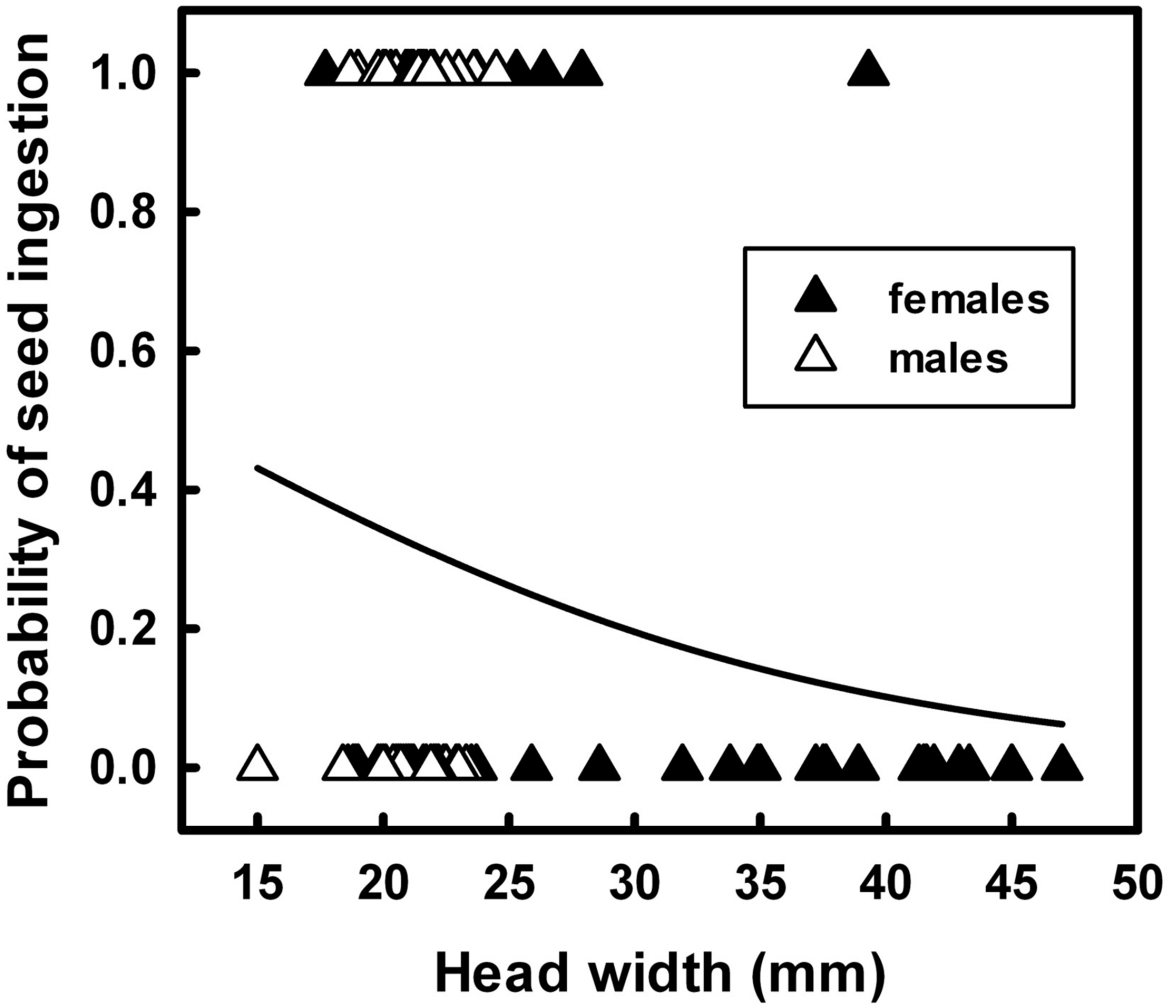
Logistic regress of ingested seeds as function of diamondback terrapin Head Width. Presence (1) or Absence (0) of ingested seeds as a function of diamondback terrapin Head Width. The curve is the probability of eelgrass seed ingestion derived from logistic regression GLM-fitted model g(1)  = e^(0.864–0.076x)^/(1+e^(0.864–0.076x)^), with α 95% CI (−1.000, 2.728) and β 95% CI (−0.156, 0.004).

Of the nine models tested, model g(1), with terrapin HW as the predictor variable, had the highest AIC_c_ weight (*w_i_* = 0.233) (Table 3; Figure 3). Model g(1) (parameter estimates: intercept  = 0.864, slope  = −0.076) predicted the probability of ingestion decreased significantly with increasing head width (Figure 3). Three other candidate models were a plausible fit though they ranked lower by AIC_c_ weight (Table 3). All three included year as an explanatory parameter.

**Table 3:** AIC-based model selection with model weights (*w_i_*) for nine candidate models to predict probability of terrapin eelgrass seed ingestion.

Model	Intercept	HW	G	Yr	HW ×G	k	ΔAICc	*w_i_*
**g(1)**	**β_0_**	**β_1_**				**2**	**0**	**0.233**
g(2)	β_0_		β_2_			2	2.493153	0.067
**g(3)**	**β_0_**			**β_3_**		**3**	**1.081933**	**0.136**
g(4)	β_0_	β_1_	β_2_			3	2.105079	0.081
**g(5)**	**β_0_**	**β_1_**		**β_3_**		**4**	**0.866103**	**0.151**
**g(6)**	**β_0_**		**β_2_**	**β_3_**		**4**	**1.321556**	**0.121**
g(7)	β_0_	β_1_	β_2_		β_4_	4	1.904298	0.090
g(8)	β_0_	β_1_	β_2_	β_3_		5	2.774923	0.058
g(9)	β_0_	β_1_	β_2_	β_3_	β_4_	6	2.664346	0.062

Seed ingested (1) or not ingested (0) was regressed (logistic regression) against terrapin head width (HW), gender (G), year captured (Yr), and the interaction between HW and G. Best-fit and plausible models in bold.

After six months in holding tanks, 11 (39.3%) of the 28 ingested seeds collected in 2010 were deemed viable, while in 2011 five (26.3%) were deemed viable. These values represent minimum estimates of viability because we assume that conditions in our holding tanks were likely to be less suitable than those in the field.

### Eelgrass shoot and seed abundance in seagrass beds

Density of SAV varied throughout the beds sampled with the highest density beds occurring along the York River and in coves along the southeast shoreline of Goodwin Island (Figure 2). The highest mean reproductive shoot density was at GSE and was nearly four times higher than that at GSW (Table 4). Seed abundance increased with shoot abundance (y = 32.8+26.1x) and differed significantly by region (*P*<<0.01). GSE had the highest mean seed abundance per m^2^ with GN a distant second (Table 4). Number of seeds produced per shoot also differed significantly by sample (*P*<<0.01) and there was a significant interaction between region and sample (*P*<0.01). Of the four models compared, model g(4), which included all the parameters, had the highest AIC *w_i_* and was the best-fit, though model g(3) with the next highest AIC_c_ *w_i_* had a higher r^2^ value than model g(4) (Tables 5 & 6). This could indicate low importance of the interaction between region and week sampled in determining reproductive shoot abundance.

**Table 4:** Estimated means (±SE) of eelgrass (*Zostera marina*) reproductive shoots, spathes, and seeds per m^2^, spathes per shoot and seeds per spathe from samples collected May 2010 at Green Point (GP) and three areas adjacent to Goodwin Islands - north (GN), southeast (GSE), and southwest (GSW),York River.

Region	Shoots	Spathes	Seeds	Spathes per Shoot	Seeds per Spathe	Est. Total Seeds Produced
GN	90.0 (12.9)	399.4 (66.1)	2050.6 (333.8)	5.1 (0.7)	5.2 (0.2)	9.4×10^8^
GSE	188.3 (26.8)	1305.9 (231.8)	6454.1 (1011.5)	7.5 (0.7)	5.1 (0.2)	1.8×10^9^
GSW	48.1 (14.3)	294.5 (78.2)	1997.7 (477.3)	5.8 (1.7)	4.8 (1.3)	2.3×10^8^
GP	58.6 (21.3)	326.4 (96.3)	2034.2 (570.6)	4.7 (1.1)	4.2 (0.8)	8.1×10^7^

Estimated seed bank by region: SAV area (ha) ×10,000 (m^2^/ha) × seeds/m^2^ SAV area (Figure 2) adapted from Orth et al. [15].

**Table 5:** AIC-based model selection for linear regression of eelgrass (*Zostera marina*) reproductive shoot abundance as a function of region (R), week sampled (W), and the interaction between R and W.

Model	Intercept	R	W	R×W	k	ΔAICc	*w_i_*	*r^2^*
g(1)	β_0_	β_1_			5	4.834352	0.064	0.288
g(2)	β_0_		β_2_		4	25.146394	0.000	0.039
**g(3)**	**β_0_**	**β_1_**	**β_2_**		**7**	**2.373648**	**0.219**	**0.439**
**g(4)**	**β_0_**	**β_1_**	**β_2_**	**β_3_**	**13**	**0**	**0.717**	**0.337**

Best-fit and plausible models in bold.

**Table 6:** Best-fit models (AIC *w_i_*≥0.2) parameter estimates and standard error of estimates from regressing reproduction shoot abundance with region, week sampled, and the interaction between region and week sampled.

Model	Parameter	Parameter Estimates	SE
g(4)	β0	3.8333	1.6055
	β1:		
	GP	−2.0000	2.2706
	GSE	2.3333	2.2706
	GSW	−2.6667	2.2706
	β2:		
	Sample 2	2.6667	2.2706
	Sample 3	0.6667	2.2706
	β3:		
	GP:Sample 2	−2.8333	3.2111
	GSE:Sample 2	6.5000	3.2111
	GSW:Sample 2	−2.0000	3.2111
	GP:Sample 3	3.8333	3.2111
	GSE:Sample 3	2.1667	3.2111
	GSW:Sample 3	3.3333	3.2111
g(3)	β0	2.917	1.234
	β1:		
	GP	−1.667	1.425
	GSE	5.222	1.425
	GSW	−2.222	1.425
	β2:		
	Sample 2	3.083	1.234
	Sample 3	2.500	1.234

Estimates of mean eelgrass seed abundance ranged from 81.0 million to 1.9 billion per region (Table 4), indicating that there are many more seeds available in the seagrass beds than are dispersed by terrapins.

### Viability and germination of field-collected seeds

The percent of viable seeds ranged across regions from 44.0% to 92.9% (Table 7). For all regions combined, the percent of viable seeds was 59%. Of the planted seeds, 35.0% and 35.7% germinated by April 2011 and April 2012, respectively.

**Table 7:** Estimate of percent viable eelgrass (*Zostera marina*) seeds from samples collected May 2010.

Region	Seed Collected	Seeds Stained	% Stained	Viable Seeds	% Viable
GN	222	25	11.3%	11	44.0%
GSE	822	82	10.0%	44	53.7%
GSW	35	14	40.0%	13	92.9%
GP	135	14	10.4%	11	78.6%

## Discussion

Finding eelgrass seeds in dried samples during processing of diamondback terrapin fecal samples in May 2009 was unexpected [13]. While it is not uncommon for aquatic turtles to ingest aquatic or terrestrial plant seeds [3], [5], [10], finding seeds from a marine angiosperm in fecal material of diamondback terrapins was unique for this estuarine species. Prior to the start of this study, there was one published record of terrapins in eelgrass beds. Radio tracked terrapins from Davis Marsh in North Carolina minimally used SAV beds in the vicinity of salt marshes [28]. There was also no mention of any plant material, terrestrial or aquatic, in the terrapin diet from that region [28]. Since finding terrapin-egested eelgrass seeds in 2009, there has been only one published account of *in situ* seed ingestion by terrapins [29]. Unfortunately, neither the type (e.g., terrestrial or aquatic) nor the species of seeds was identified; the seeds were collected from fecal samples of large female terrapins [29].

Habitat preference of small terrapins includes shallow, near-shore brackish water regions of estuaries and coastal bays [16], such as eelgrass meadows in Chesapeake Bay and the coastal bays of Virginia's Eastern Shore peninsula [14]. Fifty-five percent of all terrapins captured from May through early June were from an eelgrass bed along Green Point (Figure 2; Table 1), which included over half of the terrapins that ingested eelgrass seeds. Ninety-two percent of terrapins from Green Point were in the small size class (Table 1). Eelgrass seeds were most likely incidentally ingested while small terrapins fed on barnacles attached to eelgrass blades and spathes (seed pods), other sessile and mobile epifauna in the study sites [13]. In fecal samples, barnacles were still attached to pieces of eelgrass blades and spathes (Tulipani pers. obs.) [13]. In a related analysis, the bay barnacle *Balanus improvisus* was the most abundant species within the Green Point eelgrass bed [13]. Large female terrapins characteristically preferred deeper water of coves further away from shore, yet at times they also utilized shallower intertidal areas particularly near nesting beaches [16]. They too had ingested pieces of eelgrass [13].

The probability of seed ingestion decreased exponentially as head width increased. Small terrapins had egested all but three of the eelgrass seeds found in fecal samples over the three years. Additionally, seed ingestion by terrapins varied annually; fewer egested seeds were found in 2010 and 2011 despite increased effort to capture more terrapins during peak eelgrass seed development in May and completed seed release by mid-June [30]. Many abiotic factors affected eelgrass seed production [27] and the large-scale die-off in June 2010 likely reduced the number of seeds produced in 2011 [31], thereby decreasing the opportunity for terrapins to ingest seeds.

Seed germination rates for other aquatic turtle species vary from 7% to 83% [3], [10], which are comparable to rates for herbivorous tortoises [9], [32], [33]. For diamondback terrapins, germination rate of eelgrass seeds in a laboratory study was 14% and gut retention times ranged from 24–144 h [20], though such estimates likely vary with terrapin activity [34], [35]. Nonetheless, this study indicated that eelgrass seeds ingested by terrapins had a slightly higher potential of germination than *in situ* germination of *Zostera* seeds, which was estimated at 10% [27].

Several mark-recapture [36], [37] and tracking studies [28], [38]–[39] estimated home range size and distance traveled for diamondback terrapins. Greatest unidirectional distances (8.0–8.5 km) were always by mature females frequently traveling between marsh creeks and nesting beaches [37], [38], [40]. After being captured in a commercial gill net and transported out of the study area, one mature female from North Carolina traveled 12.5 km to return to its home area [28]. Distances for small terrapins were typically less than 1.5 km [37], [39]. In a related ultrasonic telemetry tracking project in this study area, estimated distance traveled for small terrapins was much greater than previously reported, i.e., 2.8–5.7 km based on detection records for the same male terrapin at numerous receivers for 2-d and 10-d periods [13]. Terrapins tracked in that study had preferred home areas similar in size, i.e., 50–455 ha, to terrapins in North Carolina [28], yet also engaged in occasional long-distance forays around the Goodwin Islands-Green Point region [13]. Hence, potential dispersal distances by small diamondback terrapins are much greater than previously assumed [20]. Combining greater travel distances with their characteristically staunch home range fidelity [36], terrapins have the ability to routinely transfer ingested eelgrass seeds between completely isolated eelgrass beds (Figure 2) [13].

Based on the times when seeds were found in terrapin fecal material, terrapins ingested eelgrass seeds directly from the reproductive shoots, with some seeds still found in the spathe (Tulipani, pers. obs.). No seeds were found in fecal samples from terrapins captured after mid-June when annual seed release to sediments finished [30], though pieces of eelgrass blades continued to be egested throughout the collection period ending in August each year. In eelgrass beds, 80% of seeds were retained within the bed of origin with in-sediment viability decreasing from 42% to less than 5% within 6 months [26]. By directly ingesting them from the plant, terrapins were likely to consume seeds at peak viability before they became part of the in-sediment seed bank.

To estimate number of terrapin dispersers and number of seeds potentially dispersed by terrapins for any day from mid-May through early June, we used a 2008 terrapin population estimate for Goodwin Islands [41], the sex-ratio from a related terrapin diet study [13], and the percentage of terrapins with seeds by size class and average number of seeds per terrapin (this study; Table 8). The estimated number of potential terrapin eelgrass seed dispersers ranged from 559 to 799 dispersing between 1,341 and 1,677 seeds (Table 8). Because of temporal and spatial variability in available seeds [42], the potential number of dispersed seeds would vary as well. While these dispersal estimates are an extremely small fraction of estimated total seeds produced in the Goodwin Islands-Green Point region, it may represent a significant mechanism for dispersal of viable seeds beyond the bed of origin.

**Table 8:** Estimated number of small and large terrapins potentially dispersing seeds in the Goodwin Island region.

		Low			High	
2008 population estimate (Rook 2009)		2,000			2,500	
Size Class	m	sf	Lf	m	sf	Lf
Sex ratio (m:f): 1.6∶1 (Tulipani 2013)	1,250	375	375	1,563	469	468
	Small		Large	Small		Large
Total individuals	1,625		375	2,032		468
Percent with seeds:	33%		6%	33%		6%
	536		23	671		28
		All			All	
Est. number of dispersers		559			799	
Est. seeds dispersed		1,341			1,677	

Amounts were rounded to nearest whole number. Small: males (m) and small females (sf); Large: large females (Lf); estimated mean seeds/terrapin  = 2.4.

In seagrass literature as recently as 2012, neither diamondback terrapins *Malaclemys terrapin* nor any other temperate species of turtle were represented as a key inhabitant (transient or permanent) of eelgrass meadows [17]–[19]. Until the findings in May 2009, terrapins were not considered potential vectors of eelgrass seed dispersal unlike its well-known predominantly tropical relative, the herbivorous green sea turtle *Chelonia mydas* [17], [19]. *Zostera marina* seed ingestion by this estuarine turtle is a novel finding with respect to biological dispersal vectors of eelgrass [19]. Supporting the hypothesis of diamondback terrapins as potential seed dispersers is the convergence of terrapin distribution, its annual active period and habitat use, and its facultative omnivory on eelgrass overlapping with distribution of extensive meadows of *Z. marina* in Chesapeake Bay. The interplay between terrapin and eelgrass habitats in lower Chesapeake Bay exemplifies a different mutualistic relationship between diamondback terrapins and their habitat. Terrapins gain an abundant food resource found in seagrass beds, which extends its habitat beyond a typical salt marsh. Expanded seed dispersal distances within and between beds could potentially increase genetic diversity in a specific region [43], providing seeds to failing eelgrass beds, and plant canopy maintenance by removal of biofouling epifauna and old leaf parts through direct foraging [13], [44]. Further investigation of the digestive system of diamondback terrapins could reveal if terrapins are also gaining nutritional benefit from eelgrass ingestion and digestion, as well as differences in intestinal microflora between those foraging in seagrass beds and those from salt marshes. Ongoing restoration of *Zostera marina* in Virginia's coastal bays and lagoons of the state's Eastern Shore peninsula has been very successful [45]. These are also areas where large populations of diamondback terrapin occur in Virginia (Tulipani unpublished data). Through direct foraging, diamondback terrapins may make a beneficial contribution to the health of *Zostera marina* seagrass beds.

## Acknowledgments

We thank the students and staff of the Marine Conservation Biology and Community Ecology labs at VIMS, especially of M. Seebo. We also thank R. Chambers, E. Hilton, M. LaMar, R. Seitz, and W. Roosenburg for their review and helpful comments throughout the project. We also thank the VIMS Seagrass Ecology lab for their cooperation and use of their experimental tanks. This paper is Contribution No. 3376 of the Virginia Institute of Marine Science, College of William & Mary.

## Supporting Information

Table S1 **Metadata for raw data of diamondback terrapin** ***in situ*** **ingestion of** ***Zostera marina*** **seeds collection from southwestern Chesapeake Bay SAV beds.** Information contained includes personnel responsible for collection, date of collection, and detailed description of column data contained in the Data S1 file.
(DOCX)

Table S2 **Metadata for raw data of** ***Zostera marina*** **collection from southwestern Chesapeake Bay SAV beds.** Information contained includes personnel responsible for collection, date of collection, and detailed description of column data contained in the Data S2 file. The area sampled for each replicate was 0.053 m^2^.
(DOCX)

Table S3 **Metadata for raw data of** ***Zostera marina*** **plant material density from southwestern Chesapeake Bay SAV beds.** Information contained includes personnel responsible for collection, date of collection, and detailed description of column data contained in the Data S3 file. The area sampled for each replicate was 0.053 m^2^.
(DOCX)

Table S4 **Estimated size of sampled** ***Zostera marina*** **beds from the Goodwin Islands in southwestern Chesapeake Bay.** Estimated area reported in m^2^ and hectare.
(DOCX)

Data S1 **Raw data of diamondback terrapin** ***in situ*** **ingestion of** ***Zostera marina*** **seeds collection from southwestern Chesapeake Bay SAV beds.** Data S1.csv (comma-separated values).
(CSV)

Data S2 **Raw data of** ***Zostera marina*** **reproductive shoots, spathes, and seeds collection from southwestern Chesapeake Bay SAV beds.** Data S2.csv (comma-separated values).
(CSV)

Data S3 **Raw data of** ***Zostera marina*** **supporting calculated density estimates for southwestern Chesapeake Bay SAV beds.** Data S3.csv (comma-separated values).
(CSV)
