# Supplementary material for: Evidence of Eelgrass (Zostera marina) Seed Dispersal by Northern Diamondback Terrapin (Malaclemys terrapin terrapin) in Lower Chesapeake Bay
Source: PLoS One. 2014 Jul 29;9(7):e103346. doi: 10.1371/journal.pone.0103346 (PMC4114747; doi:10.1371/journal.pone.0103346)
Supplement: Table S1 — Metadata for raw data of diamondback terrapin in situ ingestion of Zostera marina seeds collection from southwestern Chesapeake Bay SAV beds. Information contained includes personnel responsible for collection, date of collection, and detailed description of column data contained in the Data S1 file. (DOCX) [file pone.0103346.s001.docx]

**Table S1. Metadata for raw data of diamondback terrapin *in situ* ingestion of *Zostera marina* seeds collection from southwestern Chesapeake Bay SAV beds. Data contained in file Data S1.csv.**

| **Collected by:** | **DC Tulipani, M Seebo; Virginia Institute of Marine Science** |
| --- | --- |
| **Collected** | **May-June 2009, 2010, 2011** |
| **Column** | **Description** |
| Date | Capture date |
| Yr | Year of capture |
| loc | location of capture: AI = Allens Island, GI = Goodwin Island, GP = Green Point, BB = Browns Bay, PC = Perrin Cove |
| TID | Turtle Identification number |
| T.Wt | turtle weight (g) |
| CL | turtle straight carapace length (cm) |
| HW | turtle head width (mm) |
| Gender | m = male, f = female |
| SC | size class, s = small (HW < 30 mm), L = large (HW ≥ 30 mm) |
| num.seeds | number of seeds egested |
| p.a | present = 1; absent = 0 |
