# Supplementary material for: Evidence of Eelgrass (Zostera marina) Seed Dispersal by Northern Diamondback Terrapin (Malaclemys terrapin terrapin) in Lower Chesapeake Bay
Source: PLoS One. 2014 Jul 29;9(7):e103346. doi: 10.1371/journal.pone.0103346 (PMC4114747; doi:10.1371/journal.pone.0103346)
Supplement: Table S2 — Metadata for raw data of Zostera marina collection from southwestern Chesapeake Bay SAV beds. Information contained includes personnel responsible for collection, date of collection, and detailed description of column data contained in the Data S2 file. The area sampled for each replicate was 0.053 m2. (DOCX) [file pone.0103346.s002.docx]

**Table S2**. **Metadata for raw data of *Zostera marina* collection from southwestern Chesapeake Bay SAV beds contained in the Data S2 file. The area sampled for each replicate was 0.053 m^2^.**

| **Collected by:** | **DC Tulipani, M Seebo; Virginia Institute of Marine Science** |
| --- | --- |
| **Collected** | **May 2010** |
| **Column** | **Description** |
| date | date of sample collection |
| region | collection area; GP = Green Point, GN = Goodwin Island North, GSE = Goodwind Island Southeast, GSW = Goodwin Island Southwest |
| sample | sample number, 1-3 |
| rep | replicate, 1-6 |
| shoots | Yes = present, No = absent |
| tot.shoots | total number of reproductive shoots found in sample |
| shoots.subsamp | subsampled 5 if more than 5 found, all if less than 5 |
| seeds.subsamp | number of seeds found on reproductive shoots in subsample |
| spathe.subsamp | number of spathes found on reproductive shoots in subsample |
| shootsntank | if greater than 5 shoots found in a sample, extra were incubated in outdoor saltwater tanks |
