# Supplementary material for: Evidence of Eelgrass (Zostera marina) Seed Dispersal by Northern Diamondback Terrapin (Malaclemys terrapin terrapin) in Lower Chesapeake Bay
Source: PLoS One. 2014 Jul 29;9(7):e103346. doi: 10.1371/journal.pone.0103346 (PMC4114747; doi:10.1371/journal.pone.0103346)
Supplement: Table S3 — Metadata for raw data of Zostera marina plant material density from southwestern Chesapeake Bay SAV beds. Information contained includes personnel responsible for collection, date of collection, and detailed description of column data contained in the Data S3 file. The area sampled for each replicate was 0.053 m2. (DOCX) [file pone.0103346.s003.docx]

**Table S3**. **Metadata for raw data of *Zostera marina* plant material density from southwestern Chesapeake Bay SAV beds. The area sampled for each replicate was 0.053 m^2^. Data contained in file Data S3.csv.**

| **Column headings** | **Description** |
| --- | --- |
| Site | Collection sites: GP = Green Point, GN = Goodwin Island North, GSE = Goodwind Island Southeast, GSW = Goodwin Island Southwest |
| Sample | sample number, 1-3 |
| Replicate | replicate, 1-6 |
| SampId | sample identification, site-sample-replicate |
| Date | Collection date |
| Blades AFDW g | *Zostera* blade material Ash-free Dry Weight (g) |
| Roots AFDW g | *Zostera* root material Ash-free Dry Weight (g) |
