# Supplementary material for: Evidence of Eelgrass (Zostera marina) Seed Dispersal by Northern Diamondback Terrapin (Malaclemys terrapin terrapin) in Lower Chesapeake Bay
Source: PLoS One. 2014 Jul 29;9(7):e103346. doi: 10.1371/journal.pone.0103346 (PMC4114747; doi:10.1371/journal.pone.0103346)
Supplement: Table S4 — Estimated size of sampled Zostera marina beds from the Goodwin Islands in southwestern Chesapeake Bay. Estimated area reported in m2 and hectare. (DOCX) [file pone.0103346.s004.docx]

**Table S4. Estimated size of sampled** ***Zostera marina* beds from the Goodwin Islands in southwestern Chesapeake Bay.**

| **Location** | **Area (m^2^)** | **Area (ha)** |
| --- | --- | --- |
| Goodwin North | 459,371.9 | 45.9 |
| Goodwin Southeast | 928,755.1 | 92.9 |
| Goodwin Southwest | 117,418.1 | 11.7 |
| Green Point | 40,114.4 | 4.0 |
